# A Platform for Data-Centric, Continuous Epidemiological Analyses (EpiGraphHub): Descriptive Analysis

**DOI:** 10.2196/40554

**Published:** 2023-03-06

**Authors:** Flávio Coelho, Daniel Cardoso Portela Câmara, Eduardo Correa Araújo, Lucas Monteiro Bianchi, Ivan Ogasawara, Jyoti Dalal, Ananthu James, Jessica L Abbate, Aziza Merzouki, Izabel Cristina dos Reis, Kene David Nwosu, Olivia Keiser

**Affiliations:** 1 School of Applied Mathematics Fundação Getulio Vargas Rio de Janeiro Brazil; 2 The Global Research and Analysis for Public Health Network Geneva Switzerland; 3 Laboratório de Mosquitos Transmissores de Hematozoários Instituto Oswaldo Cruz Fundação Oswaldo Cruz Rio de Janeiro Brazil; 4 National Public Health School Oswaldo Cruz Foundation Rio de Janeiro Brazil; 5 Geomatys Montpellier France; 6 Institute of Global Health Faculty of Medicine University of Geneva Geneva Switzerland

**Keywords:** epidemiology, data analysis, disease surveillance, data science, public health, durability, accessibility, data set, public, public health, platform, data, application, decision, decision-making

## Abstract

**Background:**

Guaranteeing durability, provenance, accessibility, and trust in open data sets can be challenging for researchers and organizations that rely on public repositories of data critical for epidemiology and other health analytics. The required data repositories are often difficult to locate and may require conversion to a standard data format. Data-hosting websites may also change or become unavailable without warning. A single change to the rules in one repository can hinder updating a public dashboard reliant on data pulled from external sources. These concerns are particularly challenging at the international level, because policies on systems aimed at harmonizing health and related data are typically dictated by national governments to serve their individual needs.

**Objective:**

In this paper, we introduce a comprehensive public health data platform, EpiGraphHub, that aims to provide a single interoperable repository for open health and related data.

**Methods:**

The platform, curated by the international research community, allows secure local integration of sensitive data while facilitating the development of data-driven applications and reports for decision-makers. Its main components include centrally managed databases with fine-grained access control to data, fully automated and documented data collection and transformation, and a powerful web-based data exploration and visualization tool.

**Results:**

EpiGraphHub is already being used for hosting a growing collection of open data sets and for automating epidemiological analyses based on them. The project has also released an open-source software library with the analytical methods used in the platform.

**Conclusions:**

The platform is fully open source and open to external users. It is in active development with the goal of maximizing its value for large-scale public health studies.

## Introduction

Two years into the COVID-19 pandemic, the global health community has had to overcome new and unexpected challenges. Perhaps the biggest challenge beyond the immediate need for materials to protect against disease transmission and to test and treat patients has been providing timely and informative evidence to authorities for making effective public health decisions [1]. Ensuring the free flow of scientific and statistically accurate data on clinical and laboratory information about the epidemic remains a challenge, even for resource-rich countries. This ongoing challenge relates to the efficient and timely flow of such information to support an effective fight against viral spread [2-4]. The difficulty in dealing with this technical data tsunami comes from many factors:

- Slow infrastructure for reporting of health data—prior to this pandemic, the sharing of disease surveillance data was often slow, taking weeks or months [5,6] to reach country-level data repositories. COVID-19’s Omicron variant had spread to 89 countries within approximately one month of being detected [7].
- Insufficient testing capacity—since the pandemic’s beginning, the production of test kits has increased substantially, but the costs and the logistics for large-scale testing are still beyond reach for many countries [8].
- Lack of a common global data model for disease reporting—comparability of surveillance data sets is paramount to managing risks at a global scale [9,10].
- Lack of interoperability for data exchange between countries.

Improving the first 2 items depends strictly on country-funded infrastructure, but the last 2 items can be tackled through concerted actions by nongovernmental actors and the global health research community in general [11]. The development of an ecosystem of tools for online analysis of the surveillance data stream creates a demand for high-quality primary data, which can act as an incentive for countries to invest more in health-data monitoring infrastructure. Although we cannot directly influence countries’ decisions to make data available, we can speed up global accessibility.

In this paper, we present an initiative cofunded by the World Health Organization (WHO) to build an open-source platform for continuous epidemiological data analysis. It aims at filling multiple gaps in the current public health data analysis ecosystem while functioning as a simple-to-use and responsive tool for decision-makers. We call this platform EpiGraphHub (EGH). It provides automated data integration, cleaning, and harmonization combined with a web interface for easy data exploration and building of live, interactive dashboards. We will present a high-level overview of the platform and some key examples of its use to illustrate its applicability.

The proposed platform shares some similarities with other open data platforms, as shown in Table 1; however, it differentiates itself by providing additional features and focusing on public health data and epidemiological data analysis. The other tools and frameworks we compare EGH against (Table 1) have slightly different purposes. For instance, Our World in Data (OWID) [12] is quite similar to the EGH data aggregation platform, except it is structured as a collection of data-enriched articles. EGH used OWID as one of its data sources. Next, the Comprehensive Knowledge Archive Network (CKAN) [13] is a federated data platform mostly used for cataloging heterogeneous data resources with semantic annotation capabilities. EGH data sets could be part of a CKAN catalog. Socrata [14] is very similar in features to CKAN. Finally, Google Data Studio, also known as Looker Studio [15], is similar to EGH’s data-exploration component. It is an extremely powerful business intelligence tool that leverages the Google ecosystem for those who need to create analytical reports about any topic.

The remainder of the paper is divided into seven sections: (1) system architecture, where the architecture of the platform is described along with its design goals, (2) data collection, where the general design of the data collection module is detailed, (3) data transformation, where the set of transformations available for clean and preprocessed data sets is described, (4) data exploration and visualization, where the interactive web interface for visual analytics is presented, (5) data analyses, where the analytical methods currently implemented are described with examples, (6) application hosting, where the usability of the EGH platform as a common backend for web and mobile apps is described, and finally, (7) the Discussion section, where the platform’s relevance and applicability in the context of similar initiatives is discussed.

In order to make such continuous analyses possible, data from multiple sources must be integrated into a consistent data model before they can be used for analyses. A local storage layer is also provided to guarantee the persistence of the data used in the analyses. Moreover, for dynamic data sets (that are updated regularly), snapshots can be created, so that the exact version of a data set associated with published analyses can also be maintained.

**Table 1:** Feature comparison between EpiGraphHub and other open data platform tools. This comparison is not meant to be a complete evaluation of other tools, just a comparison of selected features of EpiGraphHub.

Feature	EpiGraphHub	Our World in Data	Comprehensive Knowledge Archive Network	Socrata	Google Data Studio
Automated, user-defined data collection	Yes	No	No	No	No
Connects to external databases	Yes	No	Partial^a^	Partial^a^	Yes
Dashboard creation	Yes	No	Partial^b^	Partial^a^	Yes
Codeless use	Yes	Yes	Yes	Yes	Yes
User-specific data processing and analysis	Yes	No	Yes	Yes	Partial
Open source	Yes	Partial	Yes	Yes	No
Cloud or local deployment	Yes	No	Yes	Yes	Partial
Integrated structured query language development environment	Yes	No	No	No	Yes
Epidemiological analysis libraries	Yes	No	No	No	No

^a^Can federate across instances.

^b^Can create “data preview” charts.

## Methods

### System Architecture

EGH, which is built upon the open-source business-intelligence platform Apache Superset [16], contains added features that make it more suitable for epidemiological data analyses, such as the ability to scale well horizontally, allowing it to serve heavy loads on a distributed computing infrastructure. The components of the platform, better detailed in the following subsections, include the development of a distributed data collection engine for epidemiologically relevant data sets, provision of data harmonization as a service for users, hosting of analytical dashboards with privileged access to EGH’s databases, and provision of analytical software libraries in Python and R optimized to make use of the data available on the platform.

**Figure 1:**
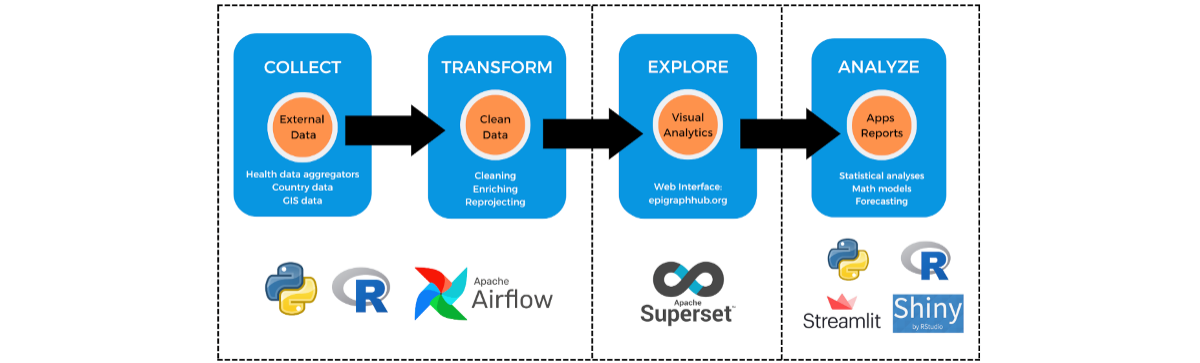
Overall organization of the EpiGraphHub platform with its 4 main modules. Data flow through the platform follows the sequence indicated by the arrows in the figure. Each data set is processed according to its specific needs, but the 4 stages above are always available. GIS: geographic information system.

**Figure 2:**
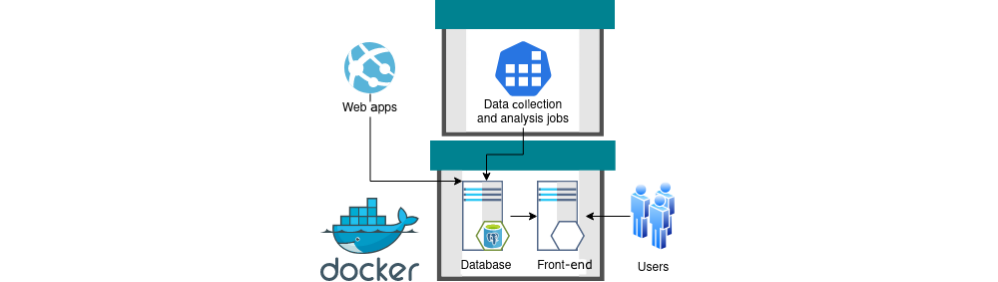
The platform architecture is based on Docker containers. In this diagram, 2 key components of the platform are represented as boxes with green lids. Containers are connected into a virtual network within the host environment and can exchange data. External web apps can connect directly to the containers to request data via the exposed application programming interface.

The platform’s architecture facilitates the automated collection, transformation, storage, and analysis and visualization of data on an entirely open-source software stack (Figure 1). The collection and transformation stages are customized for each data set and documented in the platform’s online documentation [17,18]. Portability and replication of the entire stack are important aspects of the platform’s design, which places each software service within Docker containers (Docker, Inc; Figure 2). A container is a standard unit of software that packages code and all its dependencies so the application can be deployed quickly and run reliably across different computing environments.

**Figure 3:**
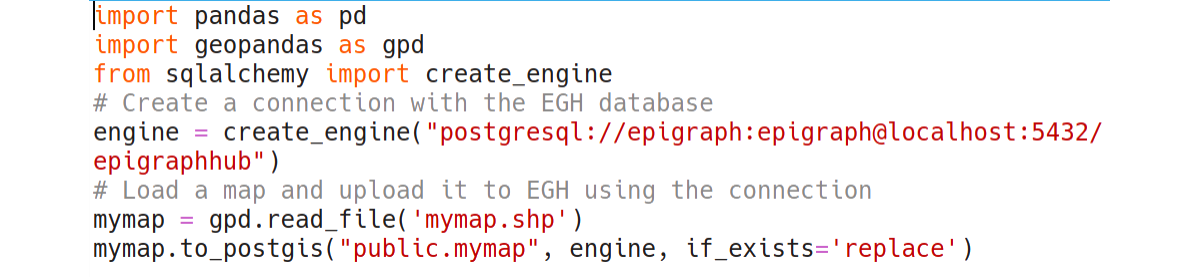
Python script to upload data to EpiGraphHub once an encrypted secure shell connection has been established.

To maximize code reusability and value for the open-source community, all the tools for data collection, transformation, and analysis were developed as EGH software libraries available both in Python and R. These libraries are fully documented and ready for independent use from the EGH platform. Figure 3 shows how a simple data upload to the platform can be accomplished with Python code.

The project provides a continuous integration (CI) workflow. Based on the GitHub action tools, CI consists of preconfigured testing scripts that run before any contribution can be merged into the main branch of our repositories. The CI routine builds all containers, checks for misconfigurations, and runs unit tests on the EGH libraries. CI is key to preventing contributions from breaking the platform. All pull requests have to pass CI tests before being merged into the main branch.

A continuous deployment (CD) workflow is currently under development to enable new releases on GitHub to automatically trigger an update on specified deployment servers. The CD workflow is an important tool to allow for the efficient delivery of new features to users.

### Data Collection

Since many health data repositories are openly accessible [19-21], the long-term availability and immutability of data sets is a key challenge when building a data analysis platform upon a broad set of data sources that operate under distinct governance systems and are funded by a variety of different sources. Lin et al [22] define the TRUST (transparency, responsibility, user focus, sustainability, and technology) principles for open data sets. Another set of principles for open data repositories are known as the FAIR (findable, accessible, interoperable, and reusable) data principles [23]. EGH follows all these principles, but also guarantees that all the data used for a particular analysis remain fixed and accessible as they were at the time the analysis was done.

In order to allow for that and if licensing terms permit, we keep a copy of the original data on our servers. In the implementation of this replication, we make a distinction between static and dynamic data sets. Static data sets are the ones not subject to updating and revision—these data sets are imported only once. Dynamic data sets, on the other hand, must be periodically updated or extended. Disease surveillance databases such as COVID-19 case counts are a good example. For these data sets, we define a periodicity for their updating, which is automatically triggered by the platform. The entire workflow related to data collection and integration is implemented using Apache Airflow [24]. Apache Airflow is a distributed task scheduler that allows for computational tasks to be defined as parts of a workflow directed acyclic graph and to be efficiently scheduled and monitored.

The criteria for selecting which data sets should be collected is first based on the relevance of our partners’ research projects, for which a collaborative partnership exists, and second on requests from our community of users. A direct example of this prioritization is the current predominance of COVID-19 data sets due to our partnership with the WHO to tackle this global challenge.

All data sets brought into the platform are fully documented with regard to their content, their provenance, and when they were collected. This allows users to ensure data quality and compare our version of the data with the versions available at their source.

**Figure 4:**
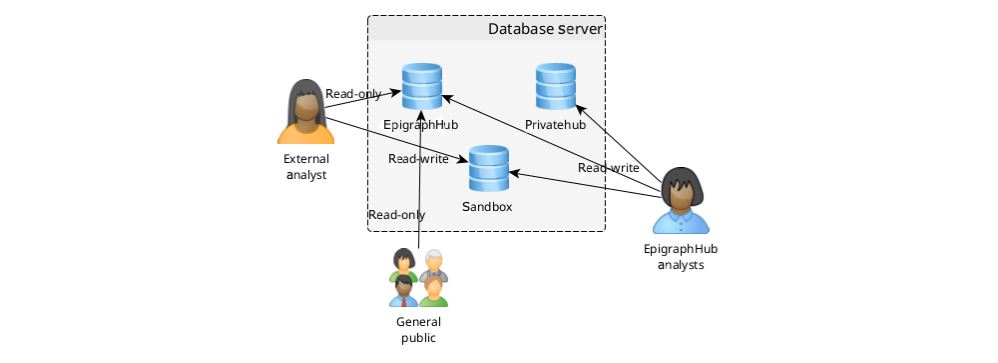
Organization of the data sets in the EpiGraphHub database server. Access control to the data sets can be configured according to the requirements of each data set.

All data sets, raw or transformed, are stored in a PostgreSQL relational database server (PostgreSQL Global Development Group) with scalable storage capacity to handle sudden growth in demand. In this database server, data are organized according to access level, wherein public and restricted-access data sets are kept in entirely separate databases (Figure 4). This guarantees the exposure of the full contents of public data sets on our web-based visual analytics tool without compromising the security of nonpublic data sets.

One key aspect of the platform is its ability to connect and pull data from widely used health information management systems (HIMS) such as the District Health Information System 2 (University of Oslo), GoData (WHO), and others. Governmental and also private HIMS are typically closed platforms where only authorized personnel have access to the data. Therefore, EGH aims to make available open-source software to facilitate pulling data from these platforms, according to the data access authorization rules from data owners. Our cloud service [25] also includes an integrated deployment of the open-source Kobo toolbox server [26] provided free of charge to partners that need to collect primary data.

The importance of the data collection feature of EGH is exemplified through its alleviation of the restrictions of data silos. This feature enables decision-makers and their data analysis teams to easily get all the relevant data for a comprehensive analysis from a single source while benefiting from our work to locate, collect, transform or clean, and store the multiple data sets.

### Data Transformation

As an integral part of the data integration pipeline, some data sets may require some transformations before they are inserted into the database for storage. This step is performed after the data collection and before the data is available in the PostgreSQL database. Examples of such transformations include the simple normalization of dates to a consistent standard or the addition of International Organization for Standardization (ISO) geocodes to spatial data sets to facilitate the generation of maps. As long as transformations can be implemented in Python or R scripts, endless transformations can be applied to the data sets. These transformations are designed to preserve the semantics of the data or their scope without altering them. Instead, they focus on enriching the data or making them consistent with data representation standards. As data collection and transformation scripts document the source of the original data and the transformations applied, all transformations are clear to users, who can modify them for personal use. The full source code for the data download and transformation processes is available in our GitHub repositories.

After the data have undergone the initial transformations and are stored in the platform, they can continue to undergo transformations through the creation of user-defined views of the database tables. Such views, which transform data before a visualization is created and do not alter the original table they pull data from, are created as structured query language (SQL) code through the web interface. These views can be shared among users and make each additional transformation completely transparent on the platform.

An example of the importance of transforming data as they are coming into the platform or at a later stage is to facilitate or enable linkage with other data sets as the data set collection on the platform grows.

The storage and sharing of views and other transformations among users can be a powerful learning tool for beginner data analysts, as well as an easy way to establish best practices for transforming and connecting data sets.

### Data Exploration and Visualization

**Figure 5:**
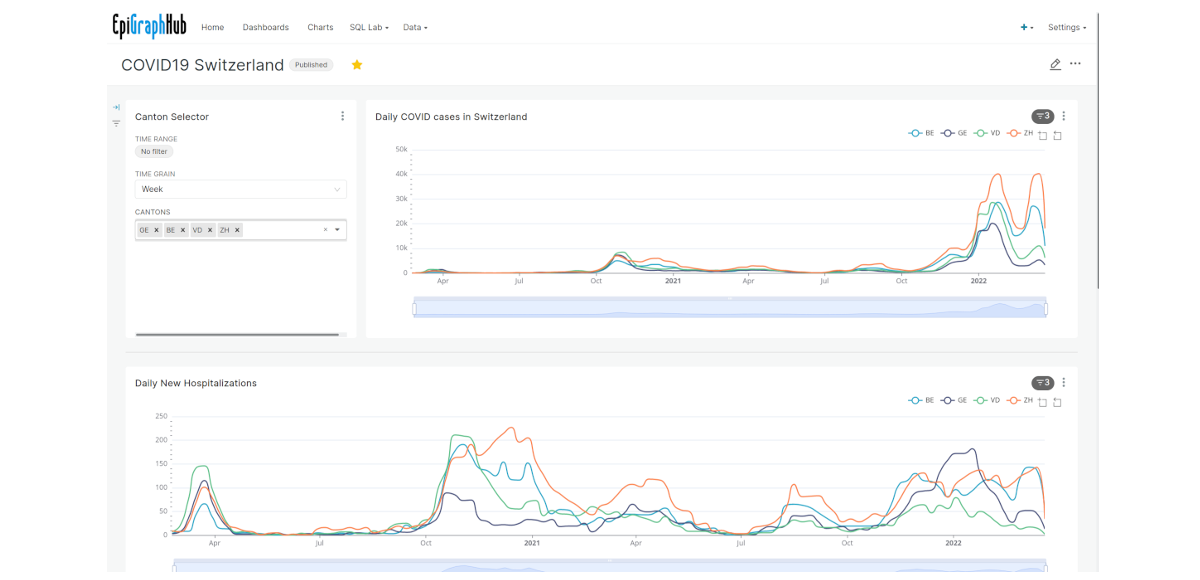
Dashboard showing COVID-19 cases and hospitalizations in Switzerland by canton, created entirely by pointing and clicking on the EpiGraphHub platform.

A web interface for interactive querying and visualization of the platform’s data sets is available [25] to help users without programming skills create visualizations from data queries. The results from the queries can then be published, shared, or integrated into live dashboards that are updated whenever the underlying data change. This interface is based on the open-source visual analytics platform Apache Superset. Figure 5 shows a dashboard created on the platform.

The sharing and publishing of the visualizations built inside the platform can be done in multiple ways. All charts, SQL queries, and dashboards created and saved by a user are given a permanent URL that can be shared with users outside the platform. Additionally, dashboards can be flagged as published, allowing all users, as well as anyone not registered on the EGH platform, to view them.

This web platform allows for fine-grained access control to data sets via customizable user permissions granted to users who have created accounts on the platform.

Another advantage of EGH’s web interface is that it offers an accessible environment for a gentle introduction to SQL through the possibility of translating point-and-click–based queries performed on the platform into the equivalent SQL code. These can be saved, shared, and further modified by users.

### Data Export and Interoperability

All the data stored in our platform can be accessed and exported by both technical and nontechnical users. Users without coding skills can use our data-exploration web interface to apply filters to the available data sets and then download or share them as JavaScript Object Notation (JSON) or comma-separated value (CSV) files. Additionally, it is possible to export the data used in each chart (including the charts in the dashboards) as a CSV file. Technically savvier users can use our Python or R libraries to access the data stored in our databases and use them outside the platform for their own projects.

Since the results of any query created in the system have a permanent URL attached, the resulting table can be referenced by external analytical environments, such as spreadsheets, R scripts, or Python scripts, or can be loaded into an external database. The same pattern holds for incorporating the charts into external web documents, allowing them to remain “live,” that is, allowing them to reflect updates in the underlying data sets. Static image snapshots of charts can also be generated at any time.

### Ethical Considerations

All the publicly available data stored in our database come from public data sources. We can also store private data sets that contain sensitive information. Such data sets will be available only for specific users in agreement with the data provider.

## Results

### Data Analyses

Different levels of analyses are possible on the EGH platform. One level is the data cleaning and transformation procedure that is applied between the collection of data and storage in the platform’s database. Another level is the analysis performed within the database through the web interface. This can be done either through the graphical interface or by writing arbitrary SQL code that runs on the server. Finally, we have the more advanced, analytical application programming interface, exposed through the EGH Python and R libraries. These software libraries expose a number of analytical methods, as well as data access functions, that help users to create their own analytical applications based on EGH’s available data.

One example of a data analysis workflow within the EGH platform was the support to the African regional office of the WHO during the first year of the COVID-19 pandemic [11,27]. Individual case data from member states were received as national line lists stored as Excel (Microsoft Corp) files. Data-quality checks were performed for each country line list, and data sets were then harmonized to have standardized variables. The following step was the integration of all data sets into the EGH database using our libraries. These data sets contained individual-level information for all countries. All variables related to the geographical localization of the cases (such as residence, place of infection, and place of reporting, among others) were then standardized using ISO 3166-1 alpha-3 codes for country, district, and province levels. These harmonization procedures were then followed by integration with data provided by the Global Administrative Areas (GADM) project [28] for all 3 administrative levels. Population data were obtained via raster files from the Gridded Population of the World (GPW; version 4) [29], which provided fine resolution for population counts inside districts and provinces of the analyzed countries. All these data-harmonization steps were followed by the development of weekly and monthly analytical reports, which included exploratory epidemiological analyses. The data sets for this project cannot be shared publicly; nonetheless, the utility of the EGH platform is not reduced, as it has been designed to host both public and restricted data sets, as described above. This allows analysts with the proper permissions to combine both public and private data sets on the same analytical report or dashboard. As the variety of analytical possibilities is quite broad, being defined by user-defined code based on our web-based tools and software libraries, we refer the reader to the documentation listed on our GitHub repositories [26] for further details.

### Application Hosting

The EGH also provides a workflow for quick deployment of web or mobile apps based on the platform. This workflow allows for CI and deployment from a GitHub repository. It currently supports Python-based Streamlit (Snowflake Inc), H20-wave (H20.ai Inc), and Python/R-based Shiny (Posit Inc) applications. Other application frameworks may be supported in the future.

Apps are encapsulated within Docker containers hosted on our server. This allows for low latency in accessing hub-hosted data sets. Docker templates are available for app developers to test and deploy their apps locally, without bothering with deployment details. Apps in production are updated via a CD workflow that allows for a rebuild of the containers whenever a new release is created in its GitHub repository. This methodology facilitates the deployment of multiple apps without overloading the platform administrators.

## Discussion

A number of health data–aggregation platforms have been developed in recent years, particularly in response to the COVID-19 pandemic. With EGH, we created a platform that, due to the transparency of each step and the minimization of the need for redoing analytical steps already performed by other users, facilitates the steps of data collection, transformation, storage, and visual exploration in a way that enhances the reproducibility of the data analysis tasks.

These data management problems are not new. In Brazil, the Infodengue and Infogripe projects have been monitoring arboviruses and influenza, respectively, and making data and epidemiological analyses open to the general public for many years [30,31]. Hürliman et al [32], in 2011, talked about the need for a global database to monitor neglected tropical diseases, but more than 10 years later, no such database exists.

During the COVID-19 pandemic, a number of new data repositories for disease surveillance were developed. For example, OWID, spearheaded by Oxford University, not only focused on disease surveillance but also played an essential role during the pandemic to guarantee an openly accessible global perspective on the spread of SARS-CoV-2 and its variants [33]. Additionally, OWID maintains live dashboards for interactive exploration of its data sets. Google, with the construction of a fine-grained collection of COVID-19 data, also entered this arena through its public data sets program [20]. Google’s data sets excel in ease of use and built-in functionality for quick visual explorations of the data.

The COVID-19 pandemic has also spurred many countries to make their own COVID-19 data available on the web. However, we suspect that this attitude will not survive the end of the pandemic. The best indication of this is the fact that the openness observed for COVID-19 data has not yet been extended to other communicable diseases. The need for more widespread transparency regarding data that is of interest to public health is evident. Data transparency should be an integral part of the governmental infrastructure for health data management and should not only occur in response to visible health emergencies. The ultimate users of EGH are, therefore, governmental and nongovernmental data creators and analysts, who should see the EGH platform as a data-agnostic hub for data that is fully automated and hassle-free. We therefore expect the platform to continue to grow by answering new demands from our user base.

The data-integration aspect of EGH goes beyond supporting open data by also facilitating effective public health policies [34]. Moreover, by being a completely open-source package that can easily be deployed in a country, it fills an important gap as an easy-to-deploy and low-cost solution for health data analysis.

Despite the benefits that the EGH platform offers, some limitations exist. The quality of the analyses performed on the platform depends on the quality of the health data on which they are based. Validating the information contained in all the data sets it aggregates is beyond the intrinsic capabilities of EGH. Therefore, to improve the quality of the produced data, we interact with partners to provide them with feedback that can enable continuous improvement in data quality at the source. Feedback from our community of analysts is a unique advantage, as it relates to the usability of the data in its current state and supports advanced analyses that are key to evidence-based policy making.

The EGH platform, although still in development, is already being used to support the COVID-19 response in many countries [11,27,35]. The Graph course platform is helping to expand our user base with its online introductory course on EGH [36]. As the pressure for urgent responses to the current pandemic finally wanes, we hope our platform will be ready to continue to provide value to disease-surveillance programs across the globe and serve as a prime example of the benefit of openly accessible public health data.

## Abbreviations

CD: continuous deployment
CI: continuous integration
CKAN: Comprehensive Knowledge Archive Network
CSV: comma-separated value
EGH: EpiGraphHub
HIMS: health information management system
ISO: International Organization for Standardization
JSON: JavaScript object notation
OWID: Our World in Data
SQL: structured query language
WHO: World Health Organization

## Data Availability

All the data stored in EpiGraphHub can be accessed through the web platform or by an encrypted secure shell connection. Anyone can create an account and download the data, but secure shell connections are restricted to the platform’s analysts.
